# ANGPTL3 overcomes sorafenib resistance via suppression of SNAI1 and CPT1A in liver cancer

**DOI:** 10.1016/j.tranon.2024.102250

**Published:** 2024-12-20

**Authors:** Yang-Hsiang Lin, Cheng-Yi Chen, Hsiang-Cheng Chi, Meng-Han Wu, Ming-Wei Lai, Chau-Ting Yeh

**Affiliations:** a Liver Research Center, Chang Gung Memorial Hospital, Linkou, Taoyuan, Taiwan; b Department of Biochemistry, College of Medicine, Chang Gung University, Taoyuan, Taiwan; c Department of Cell Biology and Anatomy, College of Medicine, National Cheng Kung University, Tainan, Taiwan; d Graduate Institute of Integrated Medicine, China Medical University, Taichung, Taiwan; e Chinese Medicine Research Center, China Medical University, Taichung, Taiwan; f Department of Pediatrics, Chang Gung Memorial Hospital, Linkou Branch and Chang Gung University College of Medicine, Taoyuan, Taiwan; g Institute of Stem Cell and Translational Cancer Research, Chang Gung Memorial Hospital, Linkou, Taoyuan, Taiwan

**Keywords:** Hepatocellular carcinoma, ANGPTL3, sorafenib, CPT1A, Protein stability

## Abstract

Liver cancer, encompassing hepatocellular carcinoma (HCC) and hepatoblastoma, the latter of which primarily occurs in early childhood, is the most common malignant tumor arising from liver and is responsible for a significant number of cancer-related deaths worldwide. Targeted drugs have been used for anti-liver cancer treatment in the advanced stage, while their efficacy is greatly compromised by development of drug resistance. Drug resistance is a complicated process regulated by intrinsic and extrinsic signals and has been associated with poorer prognosis in cancer patients. In the current study, online available dataset analysis uncovered that angiopoietin-like protein 3 (ANGPTL3) manifested lower expression in sorafenib-resistant liver cancer cell lines. Additionally, ANGPTL3 was downregulated in HCC tissues, with its expression positively correlated with good prognosis. Functionally, ectopic expression of ANGPTL3 re-sensitized sorafenib-resistant cells, enhancing the sorafenib-induced reduction in cell viability and migration by suppressing zinc finger protein SNAI1 (SNAI1) expression and the protein stability of carnitine O-palmitoyltransferase 1, liver isoform (CPT1A). Clinical correlation analysis revealed that ANGPTL3 was negatively associated with SNAI1 expression. In conclusion, we identify a novel association between *ANGPTL3, SNAI1* and *CPT1A* on sorafenib therapeutic response. Targeting ANGPTL3/SNAI1/CPT1A axis may serve as a therapeutic approach to improve prognosis of liver cancer patients with sorafenib resistance.

## Highlights

• This study identified that ANGPTL3 manifested lower expression in sorafenib-resistant liver cancer cell lines.
• Ectopic expression of ANGPTL3 re-sensitized sorafenib-resistant cells to enhance sorafenib-repressed cell viability and cell migration.
• ANGPTL3 regulated CPT1A protein stability in sorafenib-resistant cell lines.
• Targeting ANGPTL3/SNAI1/CPT1A axis may serve as a therapeutic approach for improving prognosis of HCC patients with sorafenib resistance.

## Introduction

Liver cancer, encompassing hepatocellular carcinoma (HCC) and hepatoblastoma, the latter of which primarily occurs in early childhood, is the most common malignant tumor arising in the liver [1,2]. Effective treatments for these patients can extend survival. However, development of drug resistance undermines the therapeutic efficacy. Thus, understanding the mechanisms underlying drug resistance in cancer is crucial. Sorafenib, an FDA-approved anti-cancer drug, inhibits tumor growth, angiogenesis, and metastasis by blocking multiple tyrosine kinases, including vascular endothelial growth factor receptor 2, platelet-derived growth factor receptor β, and RAF proto-oncogene serine/threonine-protein kinase [3,4]. Drug resistance is regulated by both intrinsic and extrinsic pathways [5,6], leading to poorer survival outcomes in cancer patients, including those with liver cancer. Therefore, exploring the interplay between key genes and drug resistance is essential to improve survival in liver cancer patients.

Angiopoietin-like proteins (ANGPTLs) consist of eight members, ANGPTL1–8 [7]. The structure of ANGPTL proteins is similar to that of angiopoietins, the crucial mediator for modulation of angiogenesis [8]. ANGPTL3 is mainly expressed in hepatocytes and plays a role in regulating lipoprotein lipase (*LPL*) and fatty acid oxidation [9,10]. LPL is a member of the lipase family. *LPL* functions as a key regulator in the lipolytic processing of triglyceride-rich lipoproteins, mediating lipid utilization for energy or storage [10]. Studies in human and mouse models have shown that ANGPTL3 deficiency enhances LPL activity [8], indicating that *ANGPTL3* acts as an LPL inhibitor, thereby reducing triglyceride breakdown and maintaining plasma lipid levels. Notably, under feeding conditions, ANGPTL3 interacts with ANGPTL8 to form a complex that further enhances LPL inhibition [11]. Additionally, ANGPTL3 modulates high-density lipoprotein (HDL) and low-density lipoprotein (LDL) levels, indicating that ANGPTL3 activity has some implications in cardiovascular health [12]. Ruhanen et al. demonstrated that depletion of ANGPTL3 in immortalized human hepatocyte repressed cholesterol ester synthesis [13]. Furthermore, levels of sterol O-acyltransferase 1, a key enzyme in cholesterol synthesis, were reduced in ANGPTL3-silenced cell lines, suggesting *ANGPTL3* regulates specific target gene expression. A study [14] demonstrated that ANGPTL3 was highly expressed in oral squamous cell carcinoma (OSCC), where its overexpression promoted tumor growth both *in vitro* and *in vivo* via activation of the ERK pathway. Similarly, Zhong et al. [15] demonstrated that depletion of ANGPTL3 in cervical cancer cell lines suppressed cell growth, migration and invasion. In addition, knockdown of ANGPTL3 inhibited angiogenesis in human umbilical vein endothelial cells (HUVEC) by repressing integrin αvβ3. Another investigation showed that the serum levels of ANGPTL3 were higher in HCC patients than in those with chronic hepatitis or in healthy controls [16]. Controversially, Zhao et al. [17] demonstrated that overexpression of ANGPTL3 suppressed metastasis in renal cell carcinoma (RCC) cells through modulation of vasodilator-stimulated phosphoprotein phosphorylation. Clinically, ANGPTL3 expression was lower in RCC tissues than in adjacent normal tissues. Additionally, a report showed that ANGPTL3 and ANGPTL6 expression was lower in gastric cancer (GC) tissues compared to normal gastric tissues, while ANGPTL1, ANGPTL2, and ANGPTL4 were more highly expressed in GC tissues than in normal gastric tissue [18]. Higher expression of ANGPTL1 and ANGPTL2 were associated with shorter overall survival in GC patients. These findings suggest that ANGPTL proteins have varied roles across tumor types and that ANGPTL3 may play a dual role in cancer progression. However, the functional role of *ANGPTL3* in drug resistance in liver cancer remains unclear.

In the current study, we aimed to investigate the effects of *ANGPTL3* on sorafenib response in liver cancer cell lines. Through online available dataset analysis and *in vitro* experiments, we uncovered that ANGPTL3 manifested lower expression in sorafenib-resistant liver cancer cell lines. Ectopic expression of ANGPTL3 enhanced sorafenib-repressed cell viability and cell migration via suppression of zinc finger protein SNAI1 (SNAI1) expression and the protein stability of carnitine O-palmitoyltransferase 1, liver isoform (CPT1A).

## Materials and methods

### Cell culture and reagents

Human hepatocytes (HH), which are normal liver cells, were obtained from ScienCell Research Laboratories (Carlsbad, CA, USA; catalog no 5200) and cultured in Hepatocyte Medium (Carlsbad, catalog no 5201). The hepatoblastoma-derived cell line HepG2 (RRID: CVCL_0027) [19,20] and hepatoma cell lines, including Huh7 (RRID: CVCL_0336), Hep3B (RRID: CVCL_0326), J7 (RRID: CVCL_4Z69, a gift from Dr. C.S. Yang, National Taiwan University, Taiwan [21], and Mahlavu (RRID: CVCL_0405), were cultured in Dulbecco's Modified Eagle's Medium (DMEM) supplemented with 10 % (v/v) fetal bovine serum (FBS). ANGPTL3 stable overexpression cell lines were cultured in DMEM containing 10 % (v/v) FBS with G418. All cell lines were grown at 37 °C in a humidified atmosphere of 95 % air and 5 % CO_2_. Sorafenib was purchased from Selleckchem (Houston, TX, USA, catalog no S1040). The generation of sorafenib-resistant liver cancer cell lines was performed as previously described [22]. Briefly, liver cancer cells (HepG2, Huh7, and J7) were initially cultured in DMEM supplemented with 10 % (v/v) FBS and a low concentration of sorafenib (0.5 μM). The sorafenib concentration was gradually increased with each subsequent cell passage. After several months, the sorafenib-resistant cell lines (HepG2SR, Huh7SR, and J7SR) were maintained in DMEM with 10 % (v/v) FBS and 6 μM sorafenib. Before performing *in vitro* assays, cells were tested for mycoplasma contamination (BIOTOOLS, New Taipei city, Taiwan, TTB-GBC8). Cell lines used in this study were authenticated via the Promega StemElite ID System (Promega Corporation, Madison, WI, USA)

### Quantitative reverse transcription-PCR (qRT-PCR)

To detect *ANGPTL3* and *SNAI1* expression, RNA was extracted from cells using Trizol reagent (Life Technologies Inc., Carlsbad, CA, USA, catalog no 15,596,018). The detailed qRT-PCR procedure has been described previously [23]. Briefly, qRT-PCR was performed using forward and reverse primers and 1X SYBR Green mix (Applied Biosystems, Carlsbad, CA, USA, catalog no 4,309,155). The qRT-PCR conditions were as follows: 95 °C for 10 min, followed by 40 cycles of 95 °C for 15 s and 60 °C for 1 min, with an additional dissociation step. qRT-PCR analysis was conducted using the 7500 Real-Time PCR System (Applied Biosystems). The sequences of qRT-PCR primer in this study were shown below. *ANGPTL3* forward primer: 5′- TCACAAAGCAAAAGGACACTTCA-3′; *ANGPTL3* reverse primer: 5′- CATTTAGGTTGTTTTCTCCACACTCA-3′, *SNAI1* forward primer: 5′-CCCCAATCGGAAGCCTAACT-3′; *SNAI1* reverse primer: 5′- GCTGGAAGGTAAACTCTGGATTAGA-3′; *GAPDH* forward primer: 5′- AATCCCATCACCATCTTCCA-3′; *GAPDH* reverse primer: 5′- TGGACTCCACGACGTACTCA-3′.

### Western blot analysis

The western blot procedure has been described previously [23]. Antibodies specific for ANGPTL3 (OriGene Technologies, Inc., Rockville, MD, USA, catalog no TA807389), SNAI1 (Cell Signaling Technology, Boston, MA, USA, catalog no #2532), CPT1A (Abclonal, Woburn, MA, USA, catalog no A5307) and GAPDH (Merck Millipore, Billerica, MA, USA, catalog no G8795) were used. Signal detection was performed using X-ray films or UVP ChemStudio imagers for chemiluminescence, and the intensity of each molecule was quantified using Image Gauge software. The expression levels of the target proteins were normalized to GAPDH.

### Establishment of stable ANGPTL3 overexpression and knockdown cell lines

Full-length ANGPTL3 were amplified and cloned into a pcDNA3 plasmid (Invitrogen, Carlsbad, CA, USA). Liver cancer cells were transfected with pcDNA3 (10 μg) and pcDNA3-ANGPTL3 (10 μg) via using TurboFect™ transfection reagent (Thermo Fisher Scientific, Waltham, MA, USA, catalog no R0531). A pool ANGPTL3-overexpressing cell lines were established by incubating with DMEM containing 10 % FBS and G418 (800 μg/ml). *ANGPTL3*-specific shRNAs were obtained from the National RNAi Core Facility at the Institute of Molecular Biology, Academia Sinica, Taiwan. The target sequence for ANGPTL3 shRNA was 5′-CACCCAGAAGTAACTTCACTT-3′. The sh-luc (10 μg) or ANGPTL3 shRNA plasmid (10 μg), along with the viral packaging plasmids and the viral packaging plasmids (pCMV-△R8.91 (5 μg) and pMD.G (0.5 μg)), were co-transfected into 293FT cells by TurboFect™ transfection reagent (Thermo Fisher Scientific, catalog no R0531), and the virus was collected 72 h post-transfection. Hep3B cells were infected with sh-luc or shANGPTL3 virus. A pool of ANGPTL3-depleted cell lines was established by culturing the cells in DMEM supplemented with 10 % FBS and 0.5 µg/ml puromycin.

### Cell viability assay

Cells (1 × 10^3^ /well) were seeded in 96-well plates. After treating with sorafenib, cell viability was determined using alamarBlue™ cell viability reagent (Thermo Fisher Scientific, Waltham, MA, USA, catalog no DAL1100). All values were normalized to that on day 1 and then compared to the vector control.

### Transwell migration assay

Cell migration was analyzed by *in vitro* Transwell assay (Becton-Dickinson, Franklin Lakes, NJ, USA, catalog no 3422). Cells (5 × 10⁴) incubated in serum-free medium were seeded in the upper chamber, while the lower chamber contained DMEM with 20 % FBS. After 18 h incubation, the migratory cells from the upper to the lower chamber were detected by crystal violet staining. The numbers of migratory cells were analyzed using Image J software. Meanwhile, vector control and ANGPTL3-overexpressing cells (2 × 10^6^) were seeded in 10 cm cell culture dishes with 10 % FBS DMEM. Once 90 % confluence was reached, the cells were washed three times with PBS and incubated in serum-free medium. After 48 h, conditioned media from the vector control (vc-CM) and ANGPTL3-overexpressing groups (ANGPTL3-CM) were collected, centrifuged, filtered through a 0.22 μm filter, and concentrated using a 10 kDa ultrafiltration centrifuge tube (Merck Millipore, catalog no UFC801096). The vc-CM and ANGPTL3-CM were then used to treat liver cancer cells. Cell migration in liver cancer cells was assessed using the Transwell migration assay.

### Lipid peroxidation assay

The levels of malondialdehyde (MDA) in the indicated liver cancer cells were measured by Lipid peroxidation assay kit (Abcam Inc., Waltham, MA, USA, catalog no ab233471) according to the manufacturer's instructions. In brief, standard reagent and test samples were serial diluted. Samples were added MDA color reagent (10 μl) to each well. After incubation for 30 min at room temperature, reaction solution (40 μl) was added into well. The absorbance at OD 695–700 nm was detected by the micro-plate reader.

### MG132 treatment and cycloheximide (CHX) chase experiments

For MG132 treatment assay: Before protein lysate collection, the ANGPTL3 stable cell lines were treated with MG132 (Sigma-Aldrich, St Louis, MO, USA, catalog no 474,787) for 6 h Subsequently, the protein levels of CPT1A in those cells were detected via western blot analysis. For CHX (Sigma-Aldrich, catalog no 239,763-M) chase assays, the vector control (vc) and ANGPTL3-overexpressing cell lines were treated with/without CHX for 0.5 h, 1 h and 2 h The protein levels of CPT1A were measured via western blot analysis.

### Statistical analysis

*In vitro* experimental results are presented as means ± SD from three independent experiments. Statistical analysis was conducted using SPSS software (version 20; SPSS Inc., Chicago, IL, USA). Student's *t*-test was used for comparisons between two groups, while one-way ANOVA followed by Tukey's post-hoc test was applied for comparisons among multiple groups. Survival analysis was performed using the Kaplan–Meier method with log-rank tests. P values < 0.05 were considered significant (*p < 0.01, *p* < 0.05).

## Results

### *ANGPTL3* is clinically relevant in patients with liver cancer

**Fig. 1:**
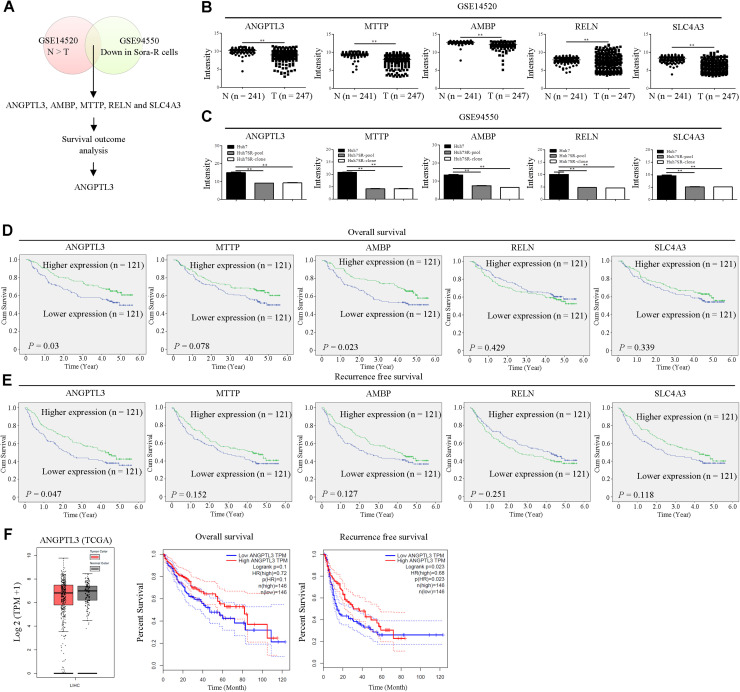
ANGPTL3 is lower expressed in HCC tissues and correlated with good prognosis in HCC patients. (A) Venn diagram of differentially expressed genes identified in two online available datasets (GSE14520 and GSE94550). Levels of ANGPTL3, MTTP, AMBP, RELN and SLC4A3 in GSE14520 (B) and GSE94550 (C) were analyzed. Kaplan-Meier analysis of overall (D) and recurrence-free survival (E) based on the indicated gene expression in GSE14520. Median expression levels of those genes were used as the cutoff. Survival was analyzed using the log-rank test. (F) Expression levels and survival outcomes of ANGPTL3 in TCGA database were analyzed using the GEPIA tool (http://gepia.cancer-pku.cn/). **, *P* < 0.01.

To identify critical genes which were involved in HCC progression and sorafenib resistance, two online available datasets (GSE14520 and GSE94550) were analyzed [24,25]. Potential target genes that were simultaneously lower expressed in HCC tissues and downregulation in sorafenib resistant cells were selected for further study, leading to the identification of *ANGPTL3*, protein AMBP (*AMBP*), microsomal triglyceride transfer protein large subunit (*MTTP*), reelin (*RELN*) and anion exchange protein 3 (*SLC4A3*) (Fig. 1A). Their expressions were lower expressed in HCC than those in the adjacent normal tissues (Fig. 1B). In addition, expression levels of those genes were also downregulated in sorafenib-resistant liver cancer cell lines (Huh7SR-pool and Huh7SR-clone) compared to HCC parental cells (Huh7) (Fig. 1C). Notably, elevated expressions of ANGPTL3 were significantly associated with better overall survival and recurrence free survival (Fig. 1D and 1E). Higher expression of AMBP was correlated with better overall survival (Fig. 1D). These findings suggested that ANGPTL3 and AMBP played the tumor suppressive role in HCC progression. To further verify these contributions, the expression levels of ANGPTL3 and its clinical significance were analyzed in TCGA dataset. Consistently, ANGPTL3 was downregulated in HCC tissues compared to adjacent normal tissues (Fig. 1F). Elevated expression of ANGPTL3 was associated with better recurrence free survival but not overall survival (Fig. 1F). Based on the consistent and collective data, *ANGPTL3* may serve as a valuable factor for evaluating the prognosis of HCC patients.

### ANGPTL3 expression is decreased in sorafenib-resistant liver cancer cell lines

**Fig. 2:**
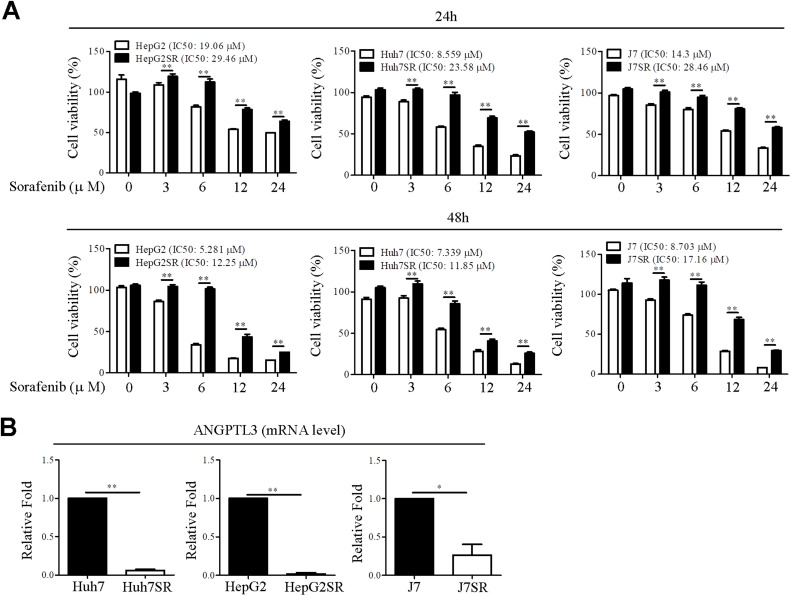
ANGPTL3 expressions are lower expressed in sorafenib-resistant liver cancer cell lines. (A) Cell viability was assayed in parental liver cancer cells- and sorafenib resistant cell lines (HepG2SR, Huh7SR and J7SR)-treated with/without sorafenib. In addition, the IC50 values of sorafenib for the indicated cell lines were shown. (B) ANGPTL3 expression was measured in liver cancer cell lines, as indicated, via qRT-PCR analysis. GAPDH was used as endogenous control.

To further confirm whether *ANGPTL3* was involved in sorafenib response, we established three sorafenib-resistant liver cancer cell lines (HepG2SR, Huh7SR and J7SR). The cell viability assay indicated that sorafenib-resistant cell lines exhibited higher survival rates following sorafenib treatment compared to their corresponding parental liver cancer cell lines (Fig. 2A). Specifically, at 24 h, the IC50 values for HepG2SR, Huh7SR, and J7SR cells were 29.46 μM (a 1.54-fold increase), 23.58 μM (a 2.75-fold increase), and 28.46 μM (a 1.99-fold increase), respectively, relative to the parental HepG2, Huh7, and J7 cells (Fig. 2A, upper panel). At 48 h, the IC50 values for HepG2SR, Huh7SR, and J7SR were 12.25 μM (a 2.32-fold increase), 11.85 μM (a 1.61-fold increase), and 17.16 μM (a 1.97-fold increase), respectively (Fig. 2A, lower panel). Compared to the parental liver cancer cell lines, expression levels of ANGPTL3 were significantly decreased in sorafenib-resistant cell lines (Fig. 2B). These observations were consistent to the dataset profiling analysis, indicating *ANGPTL3* may be involved in sorafenib response.

### Ectopic expression of ANGPTL3 enhances sorafenib-mediated effects

**Fig. 3:**
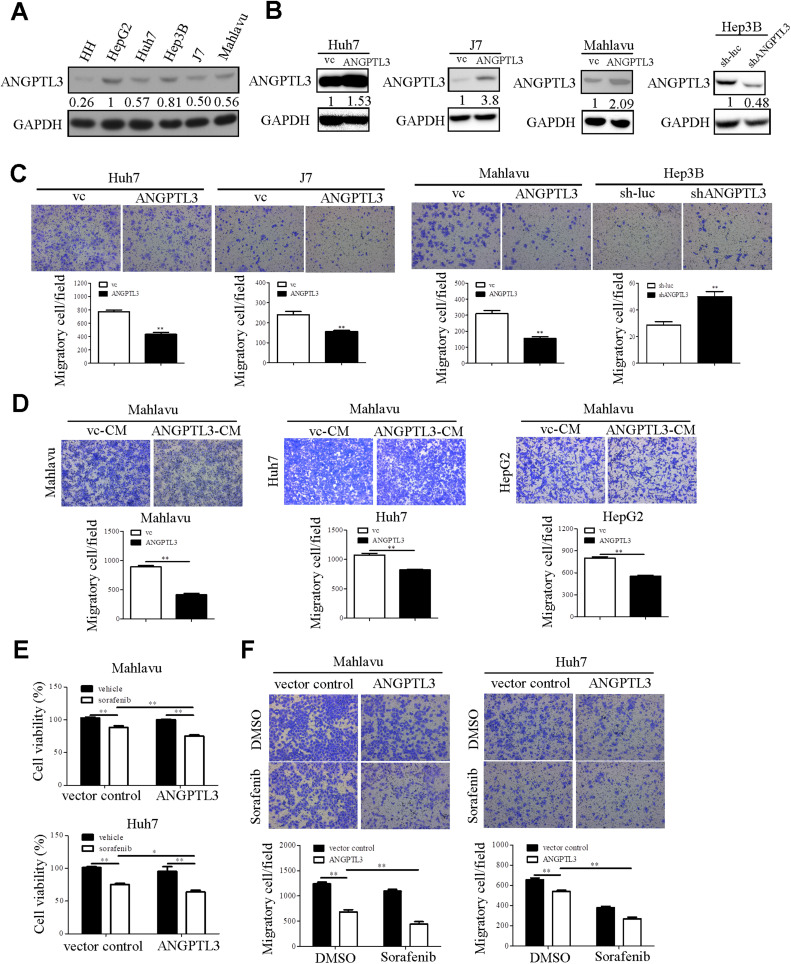
ANGPTL3 suppresses cell migration and enhanced sorafenib-induced cell death. (A) ANGPTL3 expression in human hepatocyte (HH) and liver cancer cell lines was detected by western blot analysis. GAPDH served as endogenous control. The intensity of ANGPTL3 was quantified and normalized to GAPDH, with relative fold-changes compared to those in HepG2 cells. (B) Stable overexpression of ANGPTL3 was established in Huh7, J7, and Mahlavu cell lines, while stable knockdown of ANGPTL3 was achieved in the Hep3B cell line. The expression levels of ANGPTL3 in vector control (vc) and ANGPTL3-stable cell lines were assessed by western blot analysis. The ANGPTL3 signal was quantified and normalized to GAPDH, with relative fold-changes compared to the vc group. (C) The cell migration in ANGPTL3 stable cell lines was evaluated by Transwell migration assay. The migrated cells were stained by crystal violet and quantified by Image J software. (D) Conditioned medium (CM) from ANGPTL3-overexpressing Mahlavu cell cultures was collected and used for treating liver cancer cells as indicated. The cell migration of liver cancer cells was detected by Transwell migration assay. (E) The cell viability was determined by AlamarBlue cell viability reagent in ANGPTL3-overexpressing Huh7 and Mahlavu cell lines treated with/without sorafenib (5 μM). (F) The cell motility in the indicated liver cancer cell lines was evaluated by Transwell migration assay. The migrated cells were stained by crystal violet and quantified by Image J software.

**Fig. 4:**
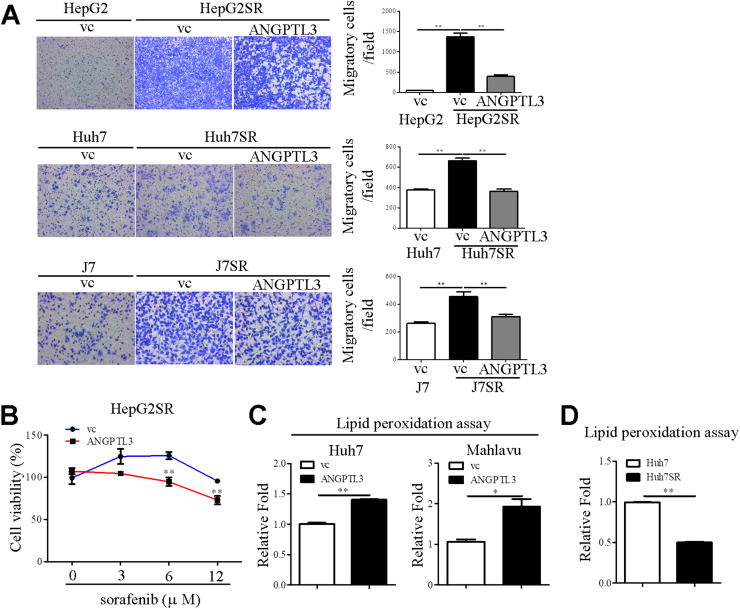
ANGPTL3 induces lipid peroxidation of liver cancer cell lines (A) The cell motility in the indicated liver cancer cell lines was evaluated by Transwell migration assay. The migrated cells were stained by crystal violet and quantified by Image J software. (B) The cell viability was determined by AlamarBlue cell viability reagent in ANGPTL3-overexpressing HepG2SR cell lines treated with/without sorafenib. The lipid peroxidation in ANGPTL3 stable cell lines (C) and sorafenib resistant cells (D) was assessed by lipid peroxidation assay. *, *P* < 0.05; **, *P* < 0.01.

So far, the effects of *ANGPTL3* on liver cancer progression and sorafenib resistance remain unclear. To elucidate the functional role of *ANGPTL3* in liver cancer cell lines, the endogenous expression of ANGPTL3 in HH and liver cancer cell lines were measured via western blot analysis. ANGPTL3 expression was lower in HH compared to other liver cancer cell lines (Fig. 3A). Based on the endogenous expression levels of ANGPTL3 in liver cancer cell lines (Fig. 3A), the stable models of ANGPTL3 overexpression and knockdown in Huh7, J7, Mahlavu and Hep3B cell lines were established. The protein levels of ANGPTL3 in cells with ANGPTL3 overexpression and knockdown were assessed using Western blot analysis (Fig. 3B). Ectopic expression of ANGPTL3 dramatically reduced cell motility compared to the control group (Fig. 3C). Conversely, knockdown of ANGPTL3 led to the opposite effects (Fig. 3C). Conditioned medium (CM) derived from cancer cells can induce a shift from an epithelial to a mesenchymal phenotype, promoting epithelial-mesenchymal transition (EMT) in the metastatic cascade via paracrine or autocrine signaling [26]. ANGPTL3 is well-known as a secreted protein [27]. To test whether extracellular ANGPTL3 regulates cell motility, conditioned media from vc-CM and ANGPTL3-CM were collected and applied to liver cancer cells to evaluate ANGPTL3-mediated cell migration. We found that treatment with ANGPTL3-CM in liver cancer cell lines (Mahlavu, Huh7, and HepG2) led to reduced cell migration (Fig. 3D), indicating that both intracellular and extracellular ANGPTL3 suppress cell motility. Furthermore, overexpression of ANGPTL3 enhanced sorafenib-repressed cell viability and cell migratory capacity (Fig. 3E and Fig. 3F). To further investigate the role of ANGPTL3 in sorafenib response, ANGPTL3-overexpressing sorafenib-resistant cell lines were established. Cell viability and motility were then assessed in these cells. The results showed that sorafenib-resistant cells exhibited higher cell migratory abilities than parental cell lines (Fig. 4A). These effects were partially blocked upon overexpression of ANGPTL3 in sorafenib-resistant cell lines (Fig. 4A). In addition, sorafenib-resistant cells with overexpression of ANGPTL3 could be sensitized to cell death (Fig. 4B). Inflammation and oxidative stress are associated with cancer progression [28]. In particular, reactive oxygen species (ROS)-mediated lipid peroxidation regulated cell viability via modulation of cell apoptosis, ferroptosis and autophagy [29]. To determine crosstalk between *ANGPTL3* and lipid peroxidation in liver cancer cell lines, lipid peroxidation assay was performed in ANGPTL3 stable cell lines. Notably, overexpression of ANGPTL3 induced lipid peroxidation production of liver cancer cell lines (Fig. 4C). On the other hand, the lipid peroxidation production was decreased in Huh7SR compared to those in Huh7 cell lines (Fig. 4D). Accordingly, we believed that *ANGPTL3* played s tumor suppressor role in regulation of cell motility, sorafenib response and lipid peroxidation of liver cancer cells.

### Overexpression of ANGPTL3 reduces SNAI1 expression

**Fig. 5:**
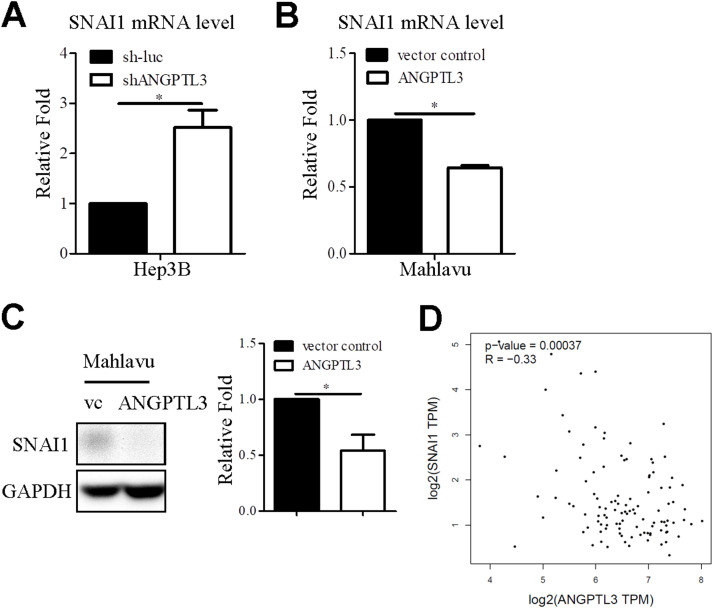
SNAI1 expression is negatively regulated by ANGPTL3 (A-B) MRNA levels of SNAI1 in ANGPTL3 stable cell lines were determined via qRT-PCR. GAPDH was used as endogenous control. (C) Protein levels of SNAI1 in the ANGPTL3-overexpressing Mahlavu cell lines were measured via western blot analysis. GAPDH was used as endogenous control. (D) The correlation between ANGPTL3 and SNAI1 in TCGA dataset was analyzed via Pearson correlation.

In fact, the metastatic feature is positively associated with drug resistance [30]. *SNAI1* is a well-known mesenchymal marker for inducing cancer cell metastasis and also associated with poor prognosis in multiple cancers [31]. We found that mRNA levels of SNAI1 were increased in ANGPTL3-depleted liver cancer cell lines (Fig. 5A). In contrast, SNAI1 expression was reduced upon overexpression of ANGPTL3 (Fig. 5B). Consistently, the protein levels of SNAI1 were significantly decreased in ANGPTL3-overexpressing Mahlavu cell lines (Fig. 5C). To support the *in vitro* findings, the correlation between *ANGPTL3* and *SNAI1* in TCGA dataset was analyzed by Pearson correlation coefficient. The results showed that ANGPTL3 expressions were significantly negatively correlated with SNAI1 expression (Fig. 5D, *r* = −0.33, *P* = 0.00037).

### Upregulation of CPT1A in sorafenib resistant cell lines is reversed in re-overexpression of ANGPTL3

**Fig. 6:**
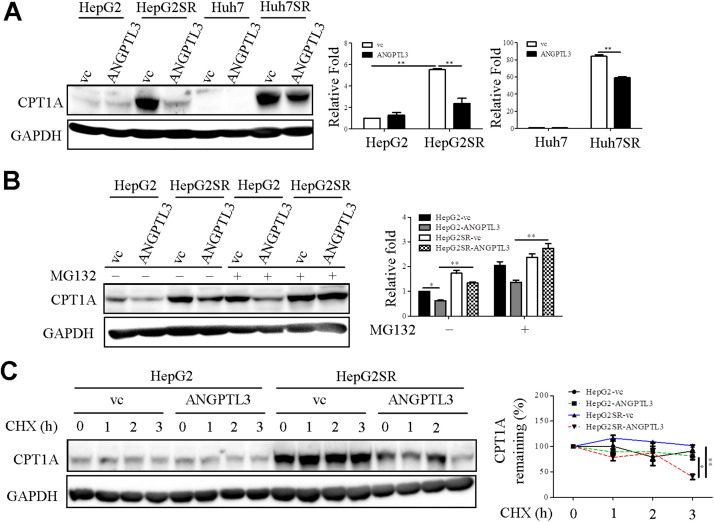
ANGPTL3 facilitates CPT1A protein degradation in sorafenib-resistant liver cancer cell lines. (A) The protein levels of CPT1A in the indicated cell lines were measured via western blot analysis. vc: vector control group. GAPDH was used as endogenous control. The fold-changes of CPT1A in the specified cell lines were quantified and presented. (B) CPT1A expression in ANGPTL3-overexpressing cell lines, as indicated, with or without MG132 treatment was detected via western blot analysis. GAPDH was used as endogenous control. The fold-changes of CPT1A in the specified cell lines were quantified and presented. (C) The turnover rate of CPT1A protein was detected in cycloheximide (CHX) chase experiments. The intensity of CPT1A was calculated and normalized to the endogenous control, GAPDH.

Increasing evidence supported that *CPT1A* gene was associated with cancer cell metastasis and drug resistance [32,33]. We found that expression levels of CPT1A were remarkable upregulated in sorafenib-resistant cell lines (Fig. 6A). These effects were partially revered upon re-overexpression of ANGPTL3 (Fig. 6A). To further characterize the underlying mechanism of *ANGPTL3*-mediated *CPT1A* expression, CPT1A protein expressions in ANPGTL3-overexpressing HepG2SR cell lines treated with/without MG132 were determined via western blot analysis. The results showed that MG132 treatment abolished ANGPTL3-repressed CPT1A protein expression in HepG2SR cell lines (Fig. 6B), suggesting that protein stability of *CPT1A* was regulated by *ANGPTL3*. To investigate this regulation further, CHX chase experiments were performed. The results revealed that overexpression of ANGPTL3 in HepG2SR cells accelerated CPT1A protein degradation (Fig. 6C). These finding indicated that ANGPTL3 regulated CPT1A protein stability in sorafenib-resistant liver cancer cell lines.

## Discussion

The crosstalk between the metastasis and drug resistance is a vital issue in deciphering the mystery of cancer progression [34,35]. The information of *ANGPTL3* on sorafenib response in liver cancer cell lines was limited. In the current study, the online available dataset (GSE94550) and *in vitro* assays demonstrated that ANGPTL3 was downregulated in sorafenib-resistant liver cancer cell lines. In addition, ectopic expression of ANGPTL3 re-sensitized sorafenib resistance via modulation of SNAI1 and CPT1A expression. Lipid metabolism plays a key role in modulating cellular functions, including cell proliferation, motility, and drug resistance [36,37]. *CPT1A* acts as a rate-limiting factor in fatty acid oxidation [38]. A previous study demonstrated that simultaneously targeting sterol O-acyltransferase 1 and CPT1A effectively suppressed tumor growth both *in vitro* and *in vivo* [39]. Sun et al. showed that ectopic expression of CPT1A enhanced chemoresistance in hypopharyngeal squamous cell carcinoma cells [40]. Mechanistically, we found that CPT1A protein stability was higher in sorafenib-resistant cell lines compared to their corresponding parental cell lines. Notably, this effect was reversed upon re-expression of ANGPTL3. This is the first evidence that *ANGPTL3* regulates CPT1A protein stability in sorafenib-resistant liver cancer cell lines. Previously, Bao and colleagues reported that ANGPTL3 expression was upregulated in sorafenib-responsive RCC patients, suggesting a positive association with favorable response [41]. Ectopic expression of ANGPTL3 also restored sorafenib sensitivity in RCC cell lines. Thus, in two distinct models (RCC and HCC), evidence supports that *ANGPTL3* functions as a tumor suppressor, modulating sorafenib-mediated cellular functions.

Previously, circulating levels of ANGPTL3 and ANGPTL4 were found to be elevated in HCC patients compared to those with chronic hepatitis and healthy individuals [16]. Conversely, another group showed that serum levels of ANGPTL3, but not ANGPTL4, was lower expressed in advanced fibrosis tissues compared to acute HCV infection. Those early studies regarding ANGPTL3 effects in HCC remain controversial. In our study, we found that ANGPTL3 RNA levels were significantly downregulated in HCC tissues compared to normal tissues. Furthermore, higher expression of ANGPTL3 was positively associated with a favorable prognosis in HCC. These findings indicate that ANGPTL3 is clinically relevant in liver cancer. Zhao et al. demonstrated that ANGPTL3 was downregulated in RCC patients compared to normal tissues [42]. Kaplan-Meier analysis showed that higher ANGPTL3 expression was positively correlated with survival outcomes. Additionally, a clinical study examined the expression levels and clinical significance of ANGPTL proteins (ANGPTL1–8) in HCC [43]. Immunohistochemical staining revealed that ANGPTL1, 3, and 4 were downregulated in HCC tissues, whereas ANGPTL2 and 5 were highly expressed in HCC. These findings are consistent with our results.

*SNAI1* and *SNAI2* are key regulators of tumor metastasis and drug resistance [44]. Previously, ANGPTL1 was shown to be downregulated in lung cancer patients compared to normal tissues [45]. Overexpression of ANGPTL1 inhibited cell motility by inducing miR-630 expression, which in turn repressed *SNAI2* expression, providing evidence that ANGPTL proteins can regulate mesenchymal marker expression. In our study, we found that ANGPTL3 overexpression suppressed SNAI1 mRNA expression, suggesting that *SNAI1* is transcriptionally regulated by *ANGPTL3*. Clinical correlation analysis further indicated that ANGPTL3 is inversely correlated with SNAI1 expression, supporting the idea that *SNAI1* is a target gene of *ANGPTL3*. The precise regulatory mechanisms between *ANGPTL3* and *SNAI1* in HCC warrant further investigation.

In conclusion, we highlight the functional role of ANGPTL3 as a key player in sorafenib resistance in HCC. Our results uncover a mechanism through which *ANGPTL3* reduces SNAI1/CPT1A expression, thereby suppressing tumor metastasis and drug resistance in liver cancer.

**List of Abbreviations:** HCC, hepatocellular carcinoma; ANGPTL3, angiopoietin-like protein 3; LPL, lipoprotein lipase; HDL, high-density lipoprotein; LDL, low-density lipoprotein; RCC, renal cell carcinoma; OSCC, oral squamous cell carcinoma; GC, gastric cancer; SNAI1, zinc finger protein SNAI1; CPT1A, carnitine O-palmitoyltransferase 1, liver isoform; HH, human hepatocytes; HUVEC, human umbilical vein endothelial cell; DMEM, Dulbecco's Modified Eagle's Medium; FBS, fetal bovine serum; qRT-PCR, quantitative reverse transcription-PCR; CM, conditioned media; MDA, malondialdehyde; CHX, cycloheximide; AMBP, protein AMBP; MTTP, microsomal triglyceride transfer protein large subunit; RELN, reelin; SLC4A3, anion exchange protein 3.

## Declarations

### Ethics approval and consent to participate

Not applicable.

## Consent for publication

Not applicable.

## Availability of data and materials

All data in this study are available upon request.

## Funding

This work was supported by grants from 10.13039/100012553Chang Gung Memorial Hospital, Taoyuan, Taiwan (CMRPG3M1271, CMRPG3M2181–2183, CRRPG3N0041, NRRPG3L6011–13, NRRPG3P0011 to YHL) and from the 10.13039/100020595National Science and Technology Council (NSTC 110–2311-B-182A-001-MY3 and NSTC 113–2311-B-182A-001- to YHL).

## CRediT authorship contribution statement

**Yang-Hsiang Lin:** Writing – review & editing, Writing – original draft, Supervision, Funding acquisition, Formal analysis, Data curation, Conceptualization. **Cheng-Yi Chen:** Formal analysis, Data curation, Conceptualization. **Hsiang-Cheng Chi:** Formal analysis, Data curation, Conceptualization. **Meng-Han Wu:** Methodology, Formal analysis, Data curation. **Ming-Wei Lai:** Formal analysis, Conceptualization. **Chau-Ting Yeh:** Writing – review & editing, Supervision, Conceptualization.

## Declaration of competing interest

The authors declare that they have no known competing financial interests or personal relationships that could have appeared to influence the work reported in this paper.

## Appendix Supplementary materials

Image, application 1
