# Supplementary figures and images for: ANGPTL3 overcomes sorafenib resistance via suppression of SNAI1 and CPT1A in liver cancer
Source: Transl Oncol. 2024 Dec 20;52:102250. doi: 10.1016/j.tranon.2024.102250 (PMC11732165; doi:10.1016/j.tranon.2024.102250)

Figure 3A

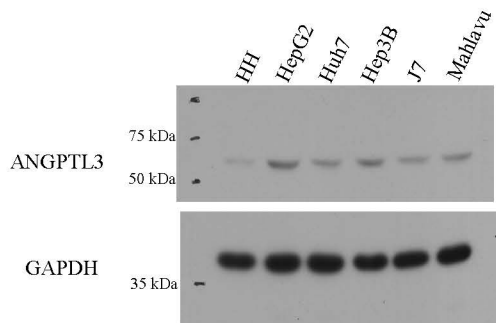

Figure 3B

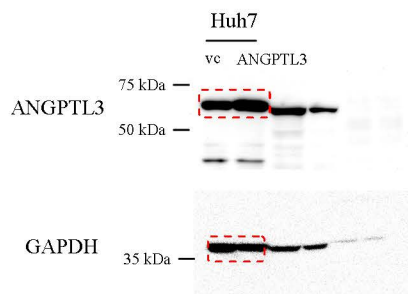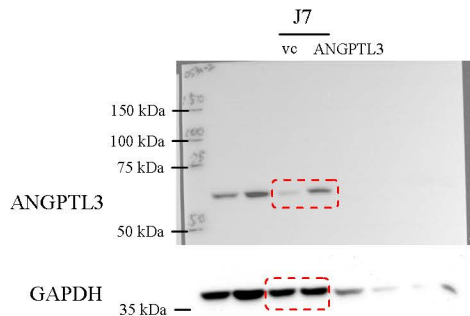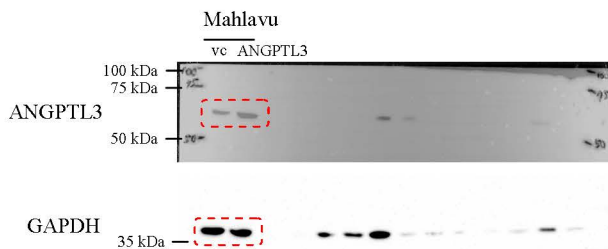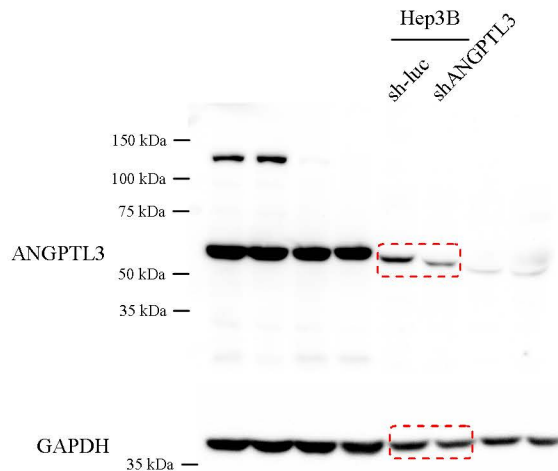

Figure 5C

SNAI1

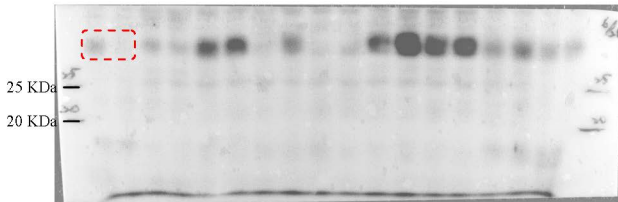

GAPDH

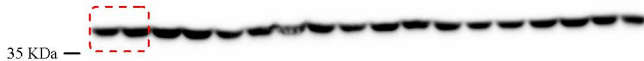

Figure 6A

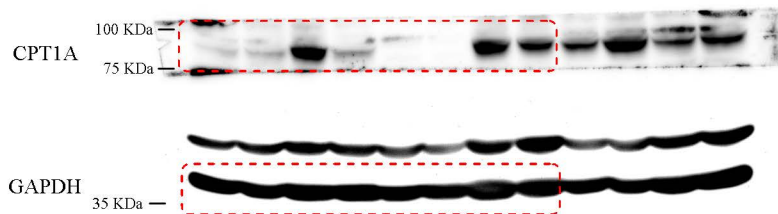

Figure 6B

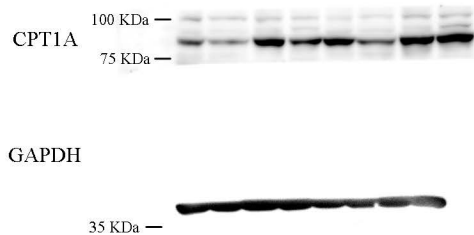

Figure 6C

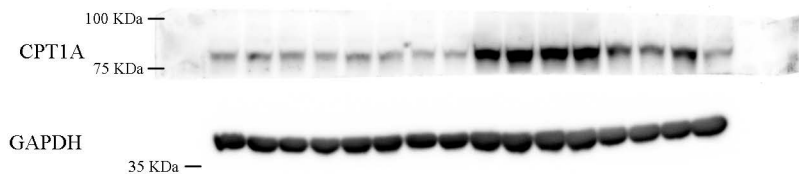

Supplement: Supplementary file 1 [file mmc1.pdf]
